# Association of Exercise and Dietary Habits With Muscle and Fat Mass in Healthy Working-Age Adults: A Cross-Sectional Study

**DOI:** 10.7759/cureus.89003

**Published:** 2025-07-29

**Authors:** Masahiro Inoue, Satoshi Yamaguchi, Naomi Ikuina, Kaori Iwakura, Chiaki Mizutani, Naoko Nomoto, Reiko Uruma, Seiji Ohtori

**Affiliations:** 1 Department of Orthopedic Surgery, Graduate School of Medicine, Chiba University, Chiba, JPN; 2 Department of Orthopedic Surgery, Graduate School of Global and Transdisciplinary Studies and College of Liberal Arts and Sciences, Chiba University, Chiba, JPN; 3 Department of Epidemiology and Public Health, Safety and Health Organization, Chiba University, Chiba, JPN; 4 Department of Clinical Nutrition, Chiba University Hospital, Chiba, JPN

**Keywords:** bioelectrical impedance analysis, body composition, body fat mass, dietary habit, dietary variety score, exercise, health checkups, lifestyle, sarcopenia, skeletal muscle mass

## Abstract

Introduction

Age-related changes in body composition, particularly sarcopenia and fat accumulation, have been widely studied in older adults, but data on healthy working-age populations remain limited. This study aimed to investigate the associations between exercise habits, dietary intake, and body composition in healthy adults aged 20-59 years.

Methods

A cross-sectional study was conducted among 1,738 employees undergoing health checkups. Body composition was assessed using bioelectrical impedance analysis (BIA). Exercise frequency and dietary habits were evaluated via questionnaire. Skeletal muscle mass index (SMI) and body fat mass index (BFMI) were calculated, and associations were analyzed by sex.

Results

SMI remained stable across age groups in men but increased with age in women; BFMI increased with age in both sexes. Regular exercise was significantly associated with higher SMI but not with BFMI. Intake of fish, soy products, and dairy products correlated with higher SMI, while higher fat intake was associated with increased BFMI.

Conclusion

Exercise habits and protein-rich diets were associated with greater skeletal muscle mass, while fat mass appeared to be more influenced by age and dietary fat intake. Further longitudinal studies are needed to clarify the causal pathways for sarcopenia prevention in working-age populations.

## Introduction

As populations age globally, there is increasing interest in preventing and/or managing age-related declines in physical function [1]. Aging induces significant changes in body composition, particularly a reduction in muscle mass and an increase in body fat mass [2]. Sarcopenia is a syndrome characterized by the loss of skeletal muscle mass and physical function; it is associated with decreased quality of life (QOL), impaired activities of daily living (ADL), increased risk of falls, and higher mortality rates [3]. Furthermore, sarcopenic obesity, a condition in which sarcopenia and obesity coexist, has been reported to have a synergistic and negative impact on health outcomes compared to sarcopenia or obesity alone [4,5].

Maintaining muscle mass and preventing excessive fat accumulation require appropriate energy intake and sufficient protein consumption [6]. Additionally, dietary variety is suggested to influence fat accumulation [7]. In recent years, numerous studies have reported the effectiveness of exercise therapy and nutritional interventions for sarcopenia, with resistance training and nutritional supplementation contributing to improvements in ADL and QOL [8,9]. However, to the best of our knowledge, the majority of these studies focus on elderly populations, and the prevalence and characteristics of sarcopenia in young adults remain insufficiently elucidated. In fact, previous reports indicate that more than 10% of young adults develop sarcopenia, regardless of race [10,11]. Sarcopenia in young adults may result from various diseases and lifestyle factors [12], highlighting the clinical importance of its early detection. Nonetheless, epidemiological data and standardized diagnostic criteria for sarcopenia in younger populations remain limited, making it difficult to accurately determine its prevalence. Although longitudinal cohort studies have suggested age- and sex-related changes in muscle mass, studies focusing exclusively on healthy adult populations are still scarce [13].

This study aimed to investigate the impact of dietary and exercise habits on muscle mass and fat mass in healthy young and middle-aged adults. We hypothesized that individuals with more frequent physical activity and healthier dietary habits would demonstrate higher skeletal muscle mass and lower fat mass. The findings of this study are expected to provide valuable insights for developing intervention strategies to promote and maintain health in young and middle-aged populations.

## Materials and methods

Study design and participants

**Figure 1:**
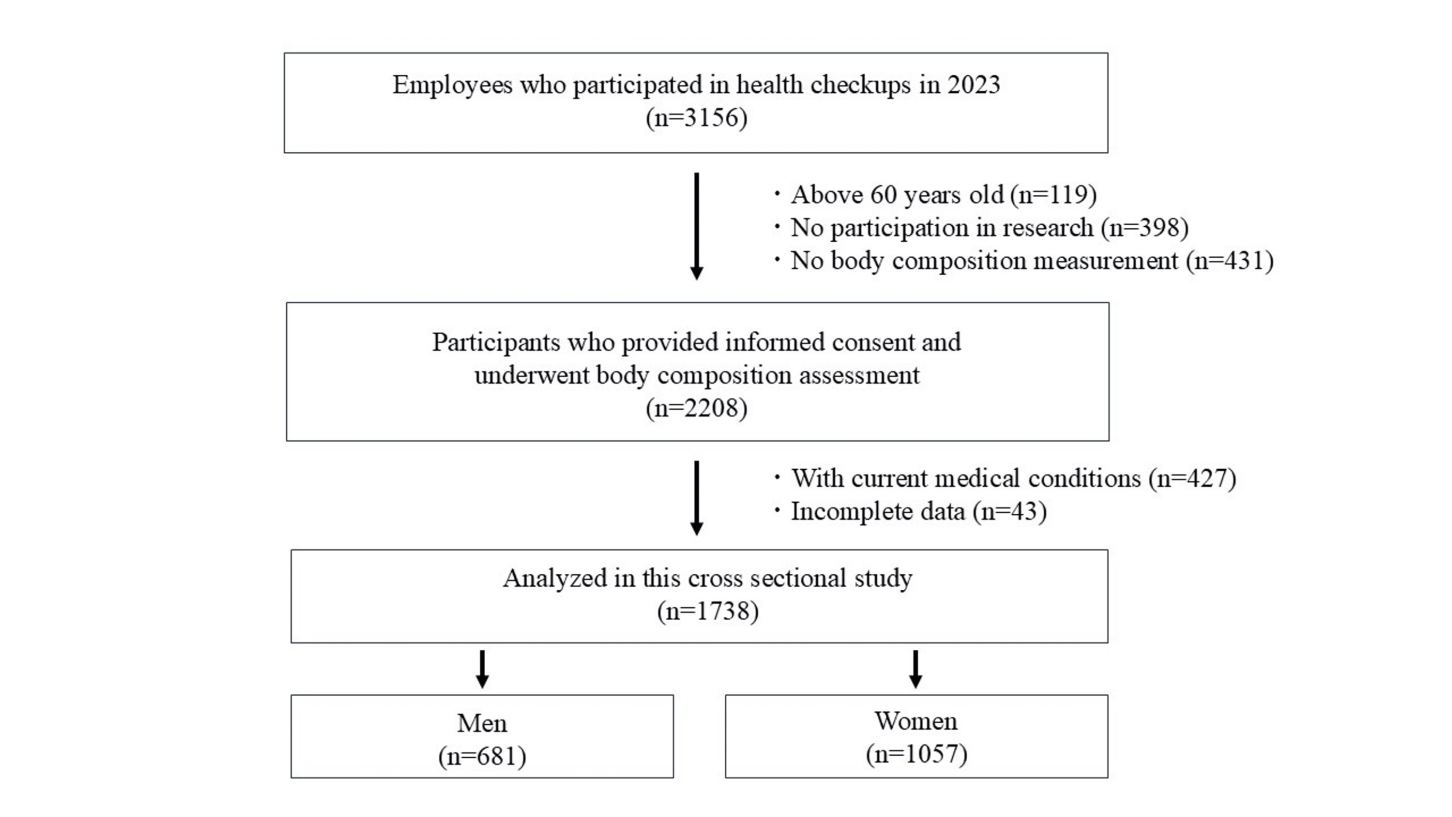
Flowchart of participant selection in this study. A flowchart illustrating the selection process of participants included in the final analysis.

This cross-sectional study was conducted at a single hospital. Among 3,156 employees who underwent routine annual health checkups in 2023, 2,305 individuals who had body composition measured and provided written informed consent were initially considered. From this group, 1,738 healthy individuals aged 20-59 years (681 men and 1,057 women; mean age, 35.7 ± 9.9 years) were included in the final analysis. Almost all participants were Japanese. The majority of participants were engaged in desk-based or light physical work. The exclusion criteria were: individuals with current medical conditions, those taking medications, those who did not provide informed consent, or those with incomplete data, as assessed using a pre-examination questionnaire (Figure 1). This study was approved by the Institutional Review Board of the participating institution.

Questionnaire on physical activity and lifestyle

Questionnaires on lifestyle and physical activity were designed to be equivalent to those used in general health examinations in Japan [14]. Before body composition measurement, participants completed a self-administered questionnaire assessing physical characteristics, lifestyle habits, and dietary intake. Collected data included age, sex, height, weight, and BMI.

Exercise habits were categorized into the following three levels: “almost none,” “sometimes,” and “almost every day.”

Body composition measurements

Body composition was assessed using bioelectrical impedance analysis (BIA) (MC-780A; TANITA, Tokyo, Japan). BIA evaluates tissue impedance by transmitting a low-level alternating current through the body, leveraging the differing impedance properties of fat, muscle, and bone. BIA is widely used in clinical settings and has been shown to correlate well with dual-energy X-ray absorptiometry in the measurement of appendicular muscle mass [15]. Measurements obtained included total body fat mass (kg) and muscle mass of the arms and legs (kg). Appendicular skeletal muscle mass (kg) was calculated as the sum of arm and leg muscle masses. Body fat mass index (BFMI) and skeletal muscle mass index (SMI) were computed using the following formulas:

BFMI (kg/m²) = total body fat mass (kg) / height² (m²)

SMI (kg/m²) = appendicular skeletal muscle mass (kg) / height² (m²) [16,17].

Sarcopenia was defined according to the criteria of the Asian Working Group for Sarcopenia, with SMI <7.0 kg/m² for men and <5.7 kg/m² for women [18].

Dietary assessment

Dietary habits were evaluated by examining the frequency of food intake. Dietary variety was measured using the Dietary Variety Score (DVS), which reflects the number of different food groups consumed over the past week. The DVS includes 10 food groups: meat, fish/shellfish, eggs, milk, soy products, green/yellow vegetables, potatoes, fruits, seaweed, and fats/oils. Participants received a score of 1 for each food group consumed “at least once every 2 days,” and 0 for food groups not consumed regularly. The total DVS ranges from 0 to 10, with higher scores indicating greater dietary variety. Based on previous studies, participants were categorized into three groups according to their DVS [7]:

Low: DVS = 0-2

Medium: DVS = 3-5

High: DVS ≥6

Statistical analysis

Given the sex-specific criteria for SMI and BFMI, analyses of the associations between body composition, participant characteristics, physical activity, and dietary factors were conducted separately for men and women. Age-related variations in SMI and BFMI were assessed using 10-year age groupings to facilitate clearer interpretation of trends. Continuous variables were compared using one-way ANOVA, followed by Tukey-Kramer’s Honestly Significant Difference (HSD) test for multiple comparisons. When normality was not assumed, the Steel-Dwass test was employed.

Variables significantly associated with SMI or BFMI in univariate analyses were further examined using multiple regression analysis to identify independent associations. Selection of potential confounders was based on references to prior literature that indicated relevance to both exposure and outcome [19].

All analyses were performed using JMP® 18 software (SAS Institute Inc., Cary, NC, USA). Data are presented as mean ± SD unless otherwise specified. A p-value <0.05 was considered statistically significant.

## Results

**Figure 2:**
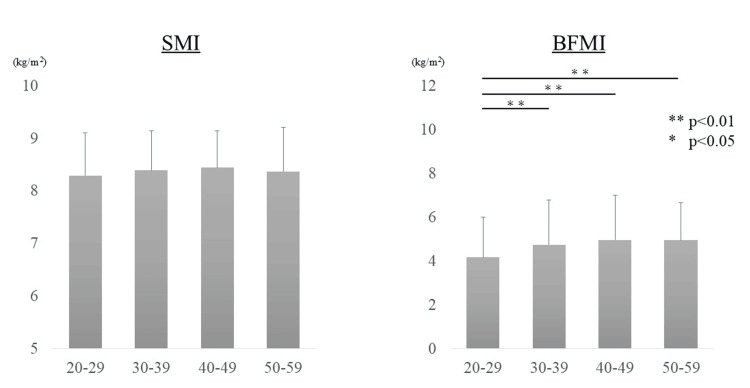
Changes in body composition in men. Age-related differences in body composition among men are presented. BFMI was significantly higher in all age groups compared to those in their 20s, suggesting that body fat mass may be associated with age. SMI: Skeletal Muscle Mass Index; BFMI: Body Fat Mass Index.

Demographic data are presented in Table 1. The mean age was 35.9 ± 8.6 years in men and 35.6 ± 10.8 years in women, with no significant difference between the two groups. The number of participants whose SMI fell below the diagnostic threshold for sarcopenia was 19 in both sexes. The mean values of SMI and BFMI by age group are as follows: among men, SMI values were 8.29 ± 0.81 in their 20s, 8.39 ± 0.75 in their 30s, 8.44 ± 0.70 in their 40s, and 8.37 ± 0.84 in their 50s. The corresponding BFMI values were 4.17 ± 1.84, 4.75 ± 2.04, 4.96 ± 2.03, and 4.95 ± 1.70, respectively. While BFMI showed a trend of increasing with age, SMI did not demonstrate significant age-related changes (Figure 2).

**Table 1:** Demographic data.

	Men	Women
No. of participants	681	1,057
Age, mean (range), year	35.9 ± 8.6	35.6 ± 10.8
Height (cm)	171.8 ± 5.9	159.0 ± 5.2
Body weight (kg)	67.9 ± 10.2	53.5 ± 9.0
BMI (kg/m²)	23.0 ± 3.1	21.1 ± 3.3
Systolic blood pressure (mmHg)	118.0 ± 14.5	108.7 ± 15.4
Skeletal muscle mass index (kg/m²)	8.37 ± 0.76	6.72 ± 0.50
Body fat mass index (kg/m²)	4.67 ± 1.98	6.13 ± 2.63
Sarcopenia, n (%)	19 (2.8%)	19 (1.8%)

**Figure 3:**
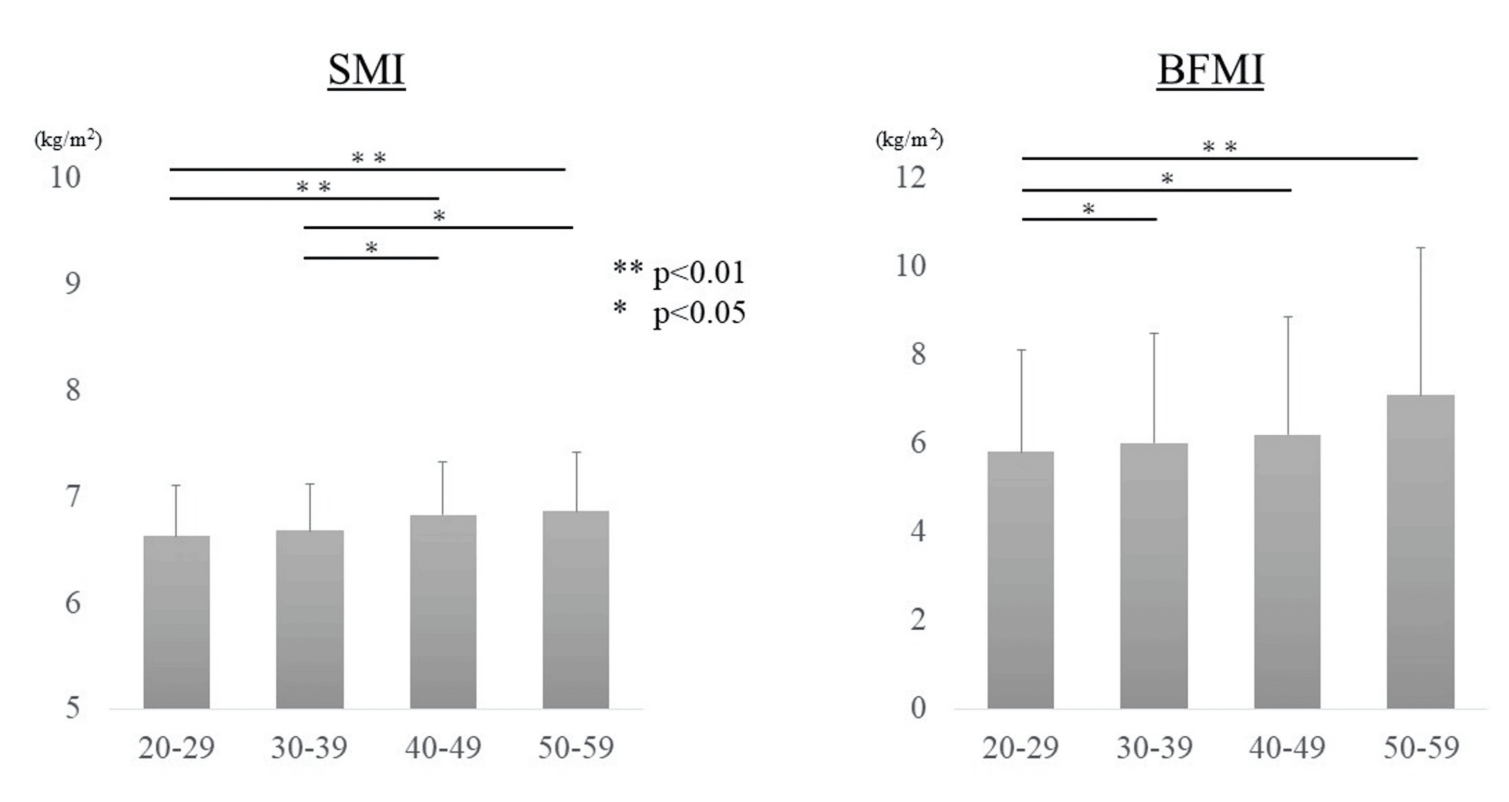
Changes in body composition in women. The association between age and body composition in women is shown. An age-related increase was observed in both SMI and BFMI. SMI: Skeletal Muscle Mass Index; BFMI: Body Fat Mass Index.

In contrast, among women, SMI values were 6.63 ± 0.48, 6.68 ± 0.44, 6.83 ± 0.50, and 6.86 ± 0.56 in the 20s, 30s, 40s, and 50s age groups, respectively. BFMI values in these groups were 5.81 ± 2.31, 6.01 ± 2.49, 6.20 ± 2.66, and 7.09 ± 3.35, respectively. Both SMI and BFMI tended to increase with age in women (Figure 3).

The physical characteristics by age group, including changes in muscle and fat mass, are summarized in Table 2. While height did not differ significantly across age groups, body weight and BMI were notably lower in participants in their 20s. Regarding the age distribution of sarcopenia, no significant difference was observed among men. However, among women, 14 of the 19 individuals classified as having sarcopenia were in their 20s, indicating a disproportionately high prevalence in this group.

**Table 2:** Physical characteristics of participants compared by age group.

	20-29	30-39	40-49	50-59	f	p
Men						
No. of patients	168	310	146	57		
Height (cm)	171.3 ± 5.8	172.0 ± 6.0	172.5 ± 5.7	170.5 ± 5.5	2.23	0.082
Body weight (kg)	65.3 ± 9.9	68.5 ± 10.4	69.6 ± 9.9	68.0 ± 9.1	5.44	0.001
BMI (kg/m²)	22.2 ± 3.0	23.1 ± 3.2	23.4 ± 3.2	23.4 ± 2.7	4.53	0.004
Sarcopenia (n)	4	8	3	4		
Women						
No. of patients	430	247	227	153		
Height (cm)	158.9 ± 5.2	159.2 ± 5.1	159.3 ± 5.5	158.5 ± 5.1	0.98	0.402
Body weight (kg)	52.2 ± 8.3	53.1 ± 8.1	54.5 ± 9.4	56.4 ± 10.7	9.75	<0.001
BMI (kg/m²)	20.6 ± 2.9	20.9 ± 3.0	21.4 ± 3.3	22.4 ± 4.0	12.9	<0.001
Sarcopenia (n)	14	2	2	1		

Regarding the association between exercise habits and body composition, individuals of both sexes who engaged in regular physical activity exhibited significantly higher SMI values. In women, SMI was significantly greater in the “Sometimes” and “Everyday” groups compared to the “Almost none” group. In men, those in the “Everyday” group demonstrated significantly higher SMI compared to both the “Almost none” and “Sometimes” groups. However, no significant association was observed between BFMI and exercise habits (Table 3).

**Table 3:** Association between exercise frequency and body composition. SMI: Skeletal Muscle Mass Index; BFMI: Body Fat Mass Index.

	SMI				BFMI		
	N	Mean ± SD	F	p	Mean ± SD	F	p
Men							
Almost none	214	8.18 ± 0.72	14.48	<0.01	4.86 ± 2.00	1.63	0.2
Sometimes	383	8.41 ± 0.77			4.61 ± 1.97		
Everyday	84	8.67 ± 0.73			4.46 ± 1.99		
Women							
Almost none	524	6.63 ± 0.51	17.65	<0.01	6.01 ± 2.70	1.29	0.28
Sometimes	466	6.78 ± 0.47			6.23 ± 2.58		
Everyday	67	6.92 ± 0.52			5.69 ± 2.44		

Analysis of dietary habits and body composition revealed that intake of fish, soy products, and dairy products was associated with higher SMI, whereas fat intake was associated with higher BFMI. In particular, food categories were significantly associated with SMI in women (Tables 4-5). An association between DVS and SMI in women was also found (Table 6). Multiple regression analysis was conducted to identify factors independently associated with SMI, including age, exercise frequency, and food items found to have significant associations in univariate analysis. The results indicated that in men, SMI was significantly associated with the presence of exercise habits. In women, both age and exercise habits were significantly associated with SMI, with higher SMI observed among those with regular exercise (Tables 7-8).

**Table 4:** Association between dietary variation and body composition in men. *Regular intake: Intake of the food item ≥ once every two days. SMI: Skeletal Muscle Mass Index; BFMI: Body Fat Mass Index.

	Regular intake*	N	SMI (mean ± SD)	t	p	BFMI (mean ± SD)	t	p
Fish/Shellfish	Yes	271	8.44 ± 0.75	1.96	0.05	4.87 ± 1.96	2.11	0.04
	No	410	8.32 ± 0.77			4.53 ± 1.99		
Meat	Yes	597	8.39 ± 0.76	1.63	0.12	4.70 ± 2.01	1.18	0.24
	No	84	8.24 ± 0.78			4.43 ± 1.78		
Eggs	Yes	407	8.41 ± 0.73	1.46	0.14	4.75 ± 1.97	1.29	0.2
	No	274	8.32 ± 0.80			4.55 ± 2.01		
Milk	Yes	264	8.41 ± 0.74	1.13	0.26	4.65 ± 1.99	-0.18	0.86
	No	417	8.35 ± 0.78			4.67 ± 1.98		
Soybean products	Yes	349	8.43 ± 0.72	2.18	0.03	4.69 ± 1.89	0.33	0.74
	No	332	8.31 ± 0.80			4.64 ± 2.08		
Vegetables	Yes	546	8.39 ± 0.76	0.93	0.35	4.71 ± 2.04	0.95	0.34
	No	135	8.32 ± 0.77			4.52 ± 1.75		
Seaweed	Yes	229	8.39 ± 0.70	0.36	0.72	4.61 ± 1.85	-0.47	0.64
	No	452	8.36 ± 0.79			4.69 ± 2.05		
Potatoes	Yes	174	8.42 ± 0.75	1.05	0.3	4.99 ± 2.15	2.45	0.02
	No	507	8.35 ± 0.76			4.56 ± 1.91		
Fruits	Yes	220	8.40 ± 0.73	0.62	0.54	4.72 ± 2.01	0.49	0.63
	No	461	8.36 ± 0.78			4.64 ± 1.97		
Fats/Oils	Yes	398	8.37 ± 0.74	-0.07	0.95	4.93 ± 2.10	4.04	<0.01
	No	283	8.37 ± 0.80			4.31 ± 1.75		

**Table 5:** Association between dietary variation and body composition in women. * Regular intake: Intake of the food item ≥ once every two days. SMI: Skeletal Muscle Mass Index; BFMI: Body Fat Mass Index.

Food Item	Regular Intake*	N	SMI (mean ± SD)	t	p	BFMI (mean ± SD)	t	p
Fish/Shellfish	Yes	308	6.74 ± 0.51	0.79	0.43	6.32 ± 2.83	1.53	0.13
	No	749	6.71 ± 0.50			6.05 ± 2.55		
Meat	Yes	852	6.71 ± 0.51	-0.54	0.59	6.15 ± 2.63	0.52	0.6
	No	205	6.73 ± 0.46			6.04 ± 2.66		
Eggs	Yes	600	6.72 ± 0.49	0.67	0.14	6.18 ± 2.72	0.69	0.49
	No	457	6.71 ± 0.51			6.06 ± 2.52		
Milk	Yes	403	6.77 ± 0.51	2.45	0.01	6.24 ± 2.89	1.07	0.28
	No	654	6.69 ± 0.49			6.06 ± 2.46		
Soybean Products	Yes	579	6.74 ± 0.49	1.5	0.14	6.16 ± 2.64	0.48	0.63
	No	478	6.69 ± 0.51			6.09 ± 2.63		
Vegetables	Yes	824	6.73 ± 0.50	1.2	0.23	6.10 ± 2.57	-0.6	0.55
	No	233	6.68 ± 0.48			6.22 ± 2.84		
Seaweed	Yes	309	6.74 ± 0.49	0.72	0.47	6.07 ± 2.54	-0.45	0.65
	No	748	6.71 ± 0.50			6.15 ± 2.67		
Potatoes	Yes	278	6.73 ± 0.51	0.63	0.53	6.26 ± 2.88	0.96	0.34
	No	779	6.71 ± 0.49			6.08 ± 2.54		
Fruits	Yes	342	6.75 ± 0.47	1.23	0.22	6.11 ± 2.53	-0.2	0.84
	No	715	6.71 ± 0.51			6.14 ± 2.68		
Fats/Oils	Yes	550	6.74 ± 0.51	1.46	0.15	6.28 ± 2.92	1.99	0.05
	No	507	6.69 ± 0.49			5.96 ± 2.28		

**Table 6:** Association between dietary variety score and body composition. SMI: Skeletal Muscle Mass Index; BFMI: Body Fat Mass Index.

	N	SMI (mean ± SD)	F	p	BFMI (mean ± SD)	F	p
Men							
Low (0-2)	464	8.33 ± 0.76	1.9	0.15	4.64 ± 1.96	0.17	0.85
Middle (3-5)	169	8.45 ± 0.79			4.75 ± 2.14		
High (>6)	48	8.46 ± 0.67			4.64 ± 1.72		
Women							
Low (0-2)	638	6.69 ± 0.51	3.75	0.03	6.15 ± 2.67	0.12	0.89
Middle (3-5)	348	6.76 ± 0.49			6.11 ± 2.59		
High (>6)	71	6.81 ± 0.47			6.00 ± 2.57		

**Table 7:** Multiple linear regression analysis of lifestyle factors associated with SMI in men. *Analysis of variance of this model. R²: Coefficient of determination; ANOVA p-value indicates overall model significance. SMI: Skeletal Muscle Mass Index.

Independent Variables	Regression Coefficient	95% CI	p	R²
			<0.01*	0.048
Intercept	8.19	-	<0.01	-
Age	0.05	-0.08 to 0.17	0.49	-
Physical activity (everyday)	0.25	0.07 to 0.43	0.01	-
Soybean products	0.04	-0.02 to 0.10	0.18	-
Fish/Shellfish	0.03	-0.03 to 0.09	0.32	-

**Table 8:** Multiple linear regression analysis of lifestyle factors associated with SMI in women. R²: coefficient of determination; SMI: Skeletal Muscle Mass Index; DVS: Dietary Variety Score. ANOVA p-value indicates overall model significance.

Independent Variables	Regression Coefficient	95% CI	p	R²
			<0.01*	0.068
Intercept	6.72	-	<0.01	-
Age	0.15	0.10 to 0.21	<0.01	-
Physical activity (almost none)	-0.1	-0.14 to -0.06	<0.01	-
Milk	-0.04	-0.07 to 0.01	0.07	-
DVS	-0.01	-0.03 to 0.03	0.98	-

## Discussion

This study investigated the influence of exercise and dietary habits on body composition, specifically skeletal muscle mass and fat mass, among healthy adults aged 20 to 59 years, based on health checkup data. The results revealed that while appendicular skeletal muscle mass did not vary significantly with age in men, it increased significantly in women from their 30s onward compared to those in their 20s. In both sexes, body fat mass increased with age. Additionally, exercise habits and intake frequencies of specific food items were found to be associated with SMI.

With regard to age-related changes, muscle mass is generally maintained until around 50 years of age and declines progressively thereafter, with an accelerated decrease observed after the age of 60 [20,21]. The median rate of muscle mass loss reported across studies is 0.47% per year in men and 0.37% per year in women, while muscle strength tends to decline at a rate two to five times faster than muscle mass [22]. In our study, no age-related decrease in muscle mass was observed in men; however, women exhibited a gradual increase in skeletal muscle mass with advancing age. In contrast, both men and women demonstrated increased fat mass with age, which is consistent with previous findings. The observed age-related increase in skeletal muscle mass among female participants may be attributed to higher body weight and BMI in the older age groups, leading to a relative increase in skeletal muscle mass.

Although sarcopenia is commonly associated with aging, several studies have reported its presence in younger populations, particularly in Asians, with prevalence rates of 7%-25% [10,23,24]. These cases may be attributed to underlying diseases or lifestyle factors. In our study, which exclusively included healthy working adults, the overall prevalence of sarcopenia was approximately 2%-3%, suggesting that sarcopenia in younger populations may be influenced by broader sociodemographic and health-related factors, including ethnicity and comorbidities.

Consistent with prior studies, our results confirmed the association between regular physical activity and increased muscle mass [25,26]. Participants of both sexes who exercised more frequently exhibited significantly higher SMI. However, we did not observe a significant relationship between exercise habits and BFMI, despite prior reports suggesting that habitual physical activity helps limit fat accumulation. This discrepancy may be due to the multifactorial nature of fat accumulation, which can be influenced by dietary patterns, metabolic status, and occupation-related physical activity. Notably, all participants in our study were working adults engaged in predominantly sedentary or light physical work, which may have limited the variability in physical activity.

Regarding dietary factors, fish, soy products, potatoes, and fats were associated with body composition, particularly in men. Prior studies have shown that DVS and the intake of protein-rich foods are positively associated with SMI [7,27]. Our findings are consistent with this, suggesting that individuals with higher SMI may consume more balanced diets. Conversely, although prior research has reported associations between high intake of processed meats, ultra-processed foods, and sugary items with increased body fat, and lower fat mass with diets rich in fruits, vegetables, and whole grains [28-30], we did not find a strong association between food categories and BFMI. However, participants with higher fat or potato intake tended to have higher BFMI, indicating a possible link between specific food types and fat accumulation.

This study has several limitations. First, the data were obtained from a single hospital, and although the majority of participants were engaged in light physical labor or desk-based work, the specific nature and duration of occupational activities were not standardized. Additionally, the sample size was relatively small, limiting the statistical power, particularly for sex-based analyses. To enhance generalizability, studies involving larger and more diverse populations are warranted. Nevertheless, all participants were non-patients and shared a similar level of occupational demand, suggesting that this dataset is valuable for evaluating body composition in healthy working individuals. Second, we were unable to include objective biochemical markers such as blood tests in this study. Future studies should incorporate such assessments to provide a more comprehensive understanding of nutritional status and metabolic health.

In conclusion, this study evaluated the associations between exercise and dietary habits with muscle and fat mass in healthy adults of working age. While causality cannot be established due to the cross-sectional design, our findings suggest that exercise habits are closely associated with muscle mass, whereas fat mass appears to be more strongly influenced by dietary factors than by physical activity alone. There is a paucity of studies focusing exclusively on healthy working-age populations, and future longitudinal research is warranted to explore how lifestyle behaviors influence the development of sarcopenia and sarcopenic obesity over time.

## Conclusions

This study examined the relationships between exercise and dietary habits and muscle and fat mass in healthy adults aged 20-59 years. The results demonstrated that both exercise and dietary habits were significantly associated with skeletal muscle mass, while fat mass was more closely related to age and dietary fat intake. Given the limited number of studies focused on healthy working-age populations, future longitudinal investigations are warranted to assess the long-term effects of lifestyle behaviors on the prevention of sarcopenia and sarcopenic obesity.
